# Gut pathogens: invaders and turncoats in a complex cosmos

**DOI:** 10.1186/1757-4749-1-3

**Published:** 2009-02-03

**Authors:** John Hermon-Taylor

**Affiliations:** 1 Division of Nutritional Sciences, Franklin-Wilkins Building, King's College London, Stamford Street, London SE1 9NH, UK

## Abstract

Intestinal infections and diarrhoeal diseases of humans are under-reported yet each account worldwide for more deaths than those from Tuberculosis. For external gut pathogens to do this they have to penetrate, survive and prosper in an established and defended ecosystem comprising the human gut with a massive immune system, a structured tight mucosal lining and a lumen densely occupied by a huge community of diverse bacteria adapted to their environment and optimised in a balanced and mutually beneficial relationship with its host.

## Commentary

Human and animal intestines are cellular ecosystems of immense complexity comprising mucosal and luminal compartments. Methodological advances particularly in high throughput DNA sequencing with its computational tools and metagenomics have allowed our explorations to probe further into the nature of the relevant microbial communities, most of whose members have never been cultured [1,2]. A study of the fecal microbiota of 60 mammalian species found the majority of sequences belonging to the Firmicutes and Bacteroides with additional Proteobacteria, Actinobacteria, Verrucomicrobia, Fusobacteria and others in diminishing proportions. There was greater similarity within host species than between species. The similarity held up in the same animal species whether in captivity or in the wild. There was close clustering by diet within herbivores, omnivores or carnivores with herbivores having the most and carnivores the fewest phyla and humans corresponded to omnivores [3].

The gut of newborn humans becomes progressively populated from the environment and converges towards the adult type over the first year [4]. In adults the intestinal microbiota mostly in the luminal compartment number in their trillions (10^14^). They form a remarkably stable community of up to about 40,000 species of bacteria with differences influenced by the genetic makeup of the host, diet, geographical location, age and the presence of inflammatory disease. The microbiota of the mucosal compartment, comprising the intestinal epithelium and surface adherent mucus layer, is necessarily smaller and significantly different from those of the lumen and faeces; it tends to be host specific, is uniform along the colon, surface fucosylated in conformity with the environment of the intestinal epithelium and less influenced by disease [5-7].

These dynamic microbial communities are controlled and ordered in co-operation and competition by quorum sensing and social recognition signalling molecules which also communicate with the host. In turn host messengers such as noradrenalin released in host stress responses as well as many other hormones, can influence the gut microbiome with effects for example on microbial growth, metabolism and expression of virulence factors [8]. Members of the gut microbiome make a substantial contribution to nutrient digestion and absorption including vitamins and micronutrients and modulate human metabolic phenotypes [9]. The intestinal microbiome by influencing the expression of host genes and interacting with epithelial cells particularly through Toll-like receptors ensures the proper post-natal maturation of the gut, stimulation of innate immune defences, the development of Peyer's patches and IgA production, angiogenesis, the education of the adaptive immune system and development of immunological tolerance [10,11].

Given the huge diversity of the microbial inhabitants of our gut the groups of external pathogens known to be able to destabilise it and cause common enteric diseases seems relatively limited. There are viruses such as Rotavirus causing severe dehydrating gastroenteritis in infants and children, enteric Adenoviruses, Noroviruses responsible for foodborne outbreaks and traveller's diarrhoea and Astroviruses. Bacteria such as *Salmonella*, *Shigella*, *Helicobacter*, *Vibrio*, *Campylobacter*, *Yersinia*, *Clostridia *and *Listeria*. Fungi such as *Candida *and *Histoplasma*. Protozoa like *Cryptosporidium*, *Giardia*, *Entamoeba *and *Cycolspora*. Helminths like Whipworm, Hookworm, Pinworm and Roundworm including *Ascaris *and *Strongyloidiasis*. The majority of these pathogens are zoonotic. Bacterial invaders have to survive the dense microbial environment of the gut and have developed multiple mechanisms for the invasion of the host [12]. The human population of Earth and the numbers of food animals needed to sustain us are both increasing. With regional and international travel together with increasing transport and trade of animals and foods, as well as spreading microbial antibiotic resistance we can expect the global burden of disease caused by gut pathogens to grow.

Common enteric diseases caused by well established external pathogens are not the only challenges we face. The enteric microbiome is a fertile environment for horizontal gene transfer [13]. Bacteria adapt and their genomes change [14,15]. Recent evidence shows that the common enteric bacterium *Escherichia coli *may display predictive behaviour on transition from the conditions of the outside world to those inside the mammalian intestine [16]. In other words they may be able to learn. These processes may be the means by which some commensal inhabitants of the gut have turned against their hosts and have evolved into pathogens. Examples include toxin producing vancomycin resistant *Enterococcus faecalis *and *E*. *coli *which can now be enteropathogenic, enterohaemorrhagic, enterotoxigenic, enteroaggregative, enteroadherent and enteroinvasive.

Inflammatory bowel diseases particularly ulcerative colitis (UC) and Crohn's disease (CD) are 'new' diseases which emerged perceptibly in Western Europe and North America in the middle of the twentieth century. They have increased in incidence and prevalence to become major healthcare and economic problems throughout Europe and North America as well as in other countries such as New Zealand and Australia. CD is generally increasing in incidence and prevalence and is now rising in former low incidence countries such as India, Korea, Japan and China. Genetic mutations conferring an increased susceptibility are clearly indentified in a proportion of people with these diseases, as they are in other chronic infections such as leprosy. The genetic data provide valuable insights into disease mechanisms.

The development of CD is known to involve one or more environmental factors. Exposure to *Mycobacterium avium *subspecies *paratuberculosis *(MAP) is a strong candidate. MAP is a multi-host intracellular pathogen which can cause systemic infection and chronic inflammation of the intestine in many species including primates. MAP infection can also persist for years without progressing to clinical disease. A recent survey by the US Department of Agriculture found that 68.1% of dairy operations in the USA are infected with MAP. These gut pathogens are transmitted to human populations in milk supplies and from sources of environmental contamination. They are difficult to detect in humans but when appropriate methods are used most people with CD are found to be infected. MAP causes a primary immune dysregulation and enteric neuropathy. The integrity of the mucosa is compromised. Segments of gross granulomatous inflammation result mostly from a perturbed neuroimmune response to the secondary penetration into the gut wall of microbiota and food residues from the gut lumen. The long term persistence of enteric MAP infection without gross inflammatory disease but with microscopic immune activation and neuropathy would perturb particularly the mucosal compartment of the gut. This would have created a distorted microenvironment conducive to the emergence of mutated *E. coli *which together with other gut microbiota act as important secondary co-pathogens in CD.

There is much to be done. A major effort in the field of modern vaccines is of great importance. The new journal 'Gut Pathogens' as an additional medium for reporting and debating the science and medicine in this field in the interests of the lives and health of all humanity is most welcome.

## Competing interests

The author currently owns the patents to a virally vectored vaccine against *Mycobacterium avium *subspecies *paratuberculosis *intended as a treatment for MAP infection in humans.
